# Periodontitis: orthodontic implications and management

**DOI:** 10.1038/s41415-024-7789-6

**Published:** 2024-09-13

**Authors:** Padhraig S. Fleming, James Andrews

**Affiliations:** 41415192378001 Chair/Professor of Orthodontics, School of Dental Science, Dublin Dental University Hospital, The University of Dublin, Trinity College Dublin, Ireland; Honorary Professor of Orthodontics, Queen Mary University of London, UK; 41415192378002 Specialist in Orthodontics, Perth, Western Australia, Australia

## Abstract

Orthodontics is increasingly ingrained in the overall management of patients with periodontitis. Advanced periodontitis is often characterised by pathological tooth migration, loss of posterior support and incisal proclination. Orthodontics may therefore offer both aesthetic and therapeutic benefit. A tailored approach to treatment, however, is necessary given the myriad of presentations and associated risk. The nuances underpinning effective treatment planning, space creation, treatment mechanics, and retention in the periodontal patient are described.

## Key points

- Advanced periodontitis may present with pathological tooth migration, loss of posterior support and incisal proclination.
- Periodontal health is generally a prerequisite to the commencement of orthodontic treatment.
- Orthodontics may provide both aesthetic and therapeutic benefit.
- Treatment planning, space creation and treatment mechanics in relation to the periodontal patient are presented.

## Introduction

The increasing accessibility to adult orthodontics allied to the closer integration of dental specialties has ensured that orthodontic treatment is increasingly ingrained in the overall management of patients with periodontitis. Advanced (Stage IV) periodontitis typically involves periodontal inflammation, attachment loss (of 5 mm or more), increased mobility, bone loss extending beyond the middle third of the root, loss of more than four teeth due to the condition and impaired masticatory function.^1^ Stage III periodontitis is associated with less tooth loss and impairment of masticatory function. However, both categories may exhibit orthodontic manifestations which may include pathological tooth migration, loss of posterior support and proclination of the incisors. As such, management of both Stage III and Stage IV periodontitis may include orthodontic treatment to improve aesthetics. Orthodontics may also offer benefits by inducing periodontal re-attachment, enhancing cleanability and improving access for instrumentation.^2^ It is noteworthy, however, that less advanced periodontitis is pervasive affecting up to 740 million people globally peaking in the fourth to fifth decades.^3^^,^^4^ As such, there is a premium on recognition, stabilisation and management of the periodontal condition during orthodontic treatment, particularly in susceptible adults. We aim to summarise the effects of periodontitis on the occlusion, the periodontal implications of orthodontics, and to highlight orthodontic considerations related to planning, space creation and mechanics in the periodontal patient.

## Pathological tooth migration

Pathological tooth migration (PTM) is a common hallmark of Stage IV periodontitis. Appropriate management is important in stabilising compromised teeth with active disease before instituting orthodontic treatment. Notwithstanding, there may be implications of ongoing disease and inappropriate management on the entire dentition.^5^ Periodontal treatment allied to supportive periodontal therapy may not lead to stabilisation and resolution of masticatory and aesthetic issues. As such, an interdisciplinary approach involving orthodontics and prosthetic management may be required in order to restore both aesthetics and function.

Orthodontic implications of periodontal breakdown can be summarised using the initialism, PERIO as follows:

- P: Proclination
- E: Extrusion
- R: Rotations
- I: Irregular spacing
- O: Overeruption.

**Fig. 1:**
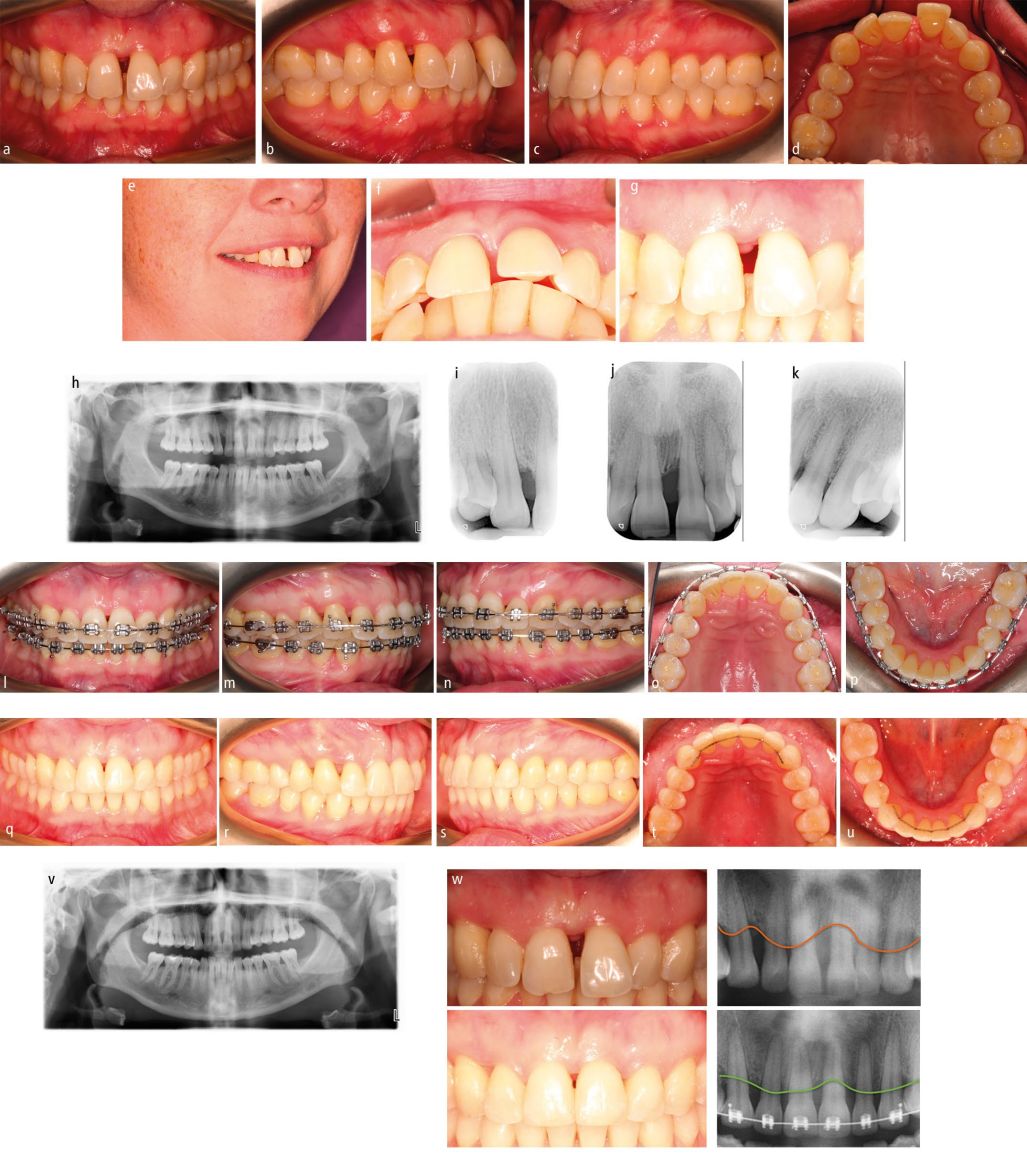
a, b, c, d, e, f, g) Proclination of the maxillary left central incisor due to a combination of reduced alveolar bone support and lip incompetence. Other orthodontic hallmarks of periodontal disease including extrusion of the upper left central incisor, local rotations and over-eruption was also present. Irregular spacing in the upper midline and distal to the upper left canine was also present. h, i, j, k) Following open flap debridement and periodontal regeneration in the upper right canine-first premolar region, fixed appliances were placed. It was hoped that the infra-boney defect in the maxillary incisor region would respond to simple debridement allied to orthodontic intrusion. l, m, n, o, p) She was treated with fixed appliances over a 6.5-month period with local interproximal reduction to reduce the dimensions of the open gingival embraces, particularly in the upper anterior region. q, r, s, t, u, v, w) A well-interdigitated Class I occlusion was obtained with the outcome retained using a combination of fixed and removable retainers. Reattachment occurred in the upper anterior region with local intrusion being favoured in order to maximise this beneficial effect of appliance therapy

Proclination of the maxillary incisors is commonly encountered due to the combined effect of reduced alveolar bone support and mismatch of soft tissue pressures between the tongue and lips.^6^ In more severe cases, this can be compounded by lip incompetence (Fig. 1), whereby pressure exerted by the tongue is unopposed by the lips and cheeks. Extrusion, rotations and local over-eruption of unopposed posterior teeth can also be prompted by a lack of alveolar bone support, while the loss or absence of opposing teeth predisposes to rapid over-eruption. Irregular spacing may relate to maturational changes and the effects of occlusion unconstrained by intact trans*-*septal fibres. This appearance may sensitise both patient and clinician to the presence of periodontal disease with occurrence in areas other than the midline being particularly symptomatic. Overall, malocclusion due to pathologic tooth migration has been observed in approximately half those with Stage III periodontitis and in over 90% of patients with Stage IV being particularly prevalent in the maxillary arch.^7^ In this study, the most prevalent manifestations included spacing (64%), extrusion (56%), and proclined incisors (32%), with Class I, II and III malocclusions affected.

## Orthodontics and periodontal support

Orthodontic treatment is reliant on bone turnover and tissue remodelling inducing osteoblastic and osteoclastic activity and inter-cellular messaging leading to aseptic inflammation.^8^ It is typically suggested that treatment should be postponed until any active periodontitis has been controlled with ongoing maintenance being undertaken in susceptible patients. In particular, untreated inflammation may predispose to the persistence or aggravation of the underlying condition. Moreover, careless planning, poorly controlled mechanics and excessive levels of intervention may predispose to local or indeed more generalised exacerbation of insipient periodontal issues.

The published primary and secondary research typically highlight limited adverse periodontal effects with a mean reduction in attachment levels of 0.11 mm being reported in a meta-analysis involving nine primary studies.^9^ As such, it could be concluded that orthodontics is almost ‘periodontally neutral'. However, it is noteworthy that orthodontics is generally undertaken on healthy periodontal conditions and good baseline levels of oral hygiene. Moreover, orthodontics typically takes place over relatively short periods ensuring that periodontitis rarely manifests. As such, a ‘non-periodontal cohort' is generally treated for relatively short periods of time. Minimal periodontal destruction is therefore typically intuitive; this does not negate the need for careful management particularly of those with periodontal susceptibility.

In contrast, orthodontic treatment is increasingly viewed as a cornerstone of the management of advanced (Stage III and Stage IV) periodontitis. In particular, Stage IV periodontitis may involve a range of orthodontic manifestations. These may dictate treatment from an aesthetic perspective. Moreover, pathological tooth migration may predispose to non-axial loading and an unstable occlusion potentially inducing further attachment loss, abnormal mobility (fremitus), and alveolar bone loss.^10^

Orthodontic treatment may lead to local reattachment with a systematic review involving 30 primary studies suggesting that combined periodontal-orthodontic treatment induced a marginal improvement in probing pocket depths (PPD), papillary height and attachment levels relative to periodontal treatment including regenerative treatments.^9^^,^^11^ Specifically, a mean attachment gain (0.24 mm), improvement in marginal bone levels (0.36 mm) and increase in papillary height (1.36 mm) was observed.^9^

## Periodontal stabilisation and maintenance

Cooperation between the orthodontist and periodontist or general dentist is important in the diagnosis and ongoing management of a periodontally-susceptible orthodontic patient. In particular, close liaison may be helpful in determining the chronology of treatment, the optimal treatment plan, in monitoring treatment progress, and in planning retention and post-treatment maintenance.^2^ Given the relatively fine balance between risk and benefit that may exist, treatment decisions may be informed by clinical and radiographic findings with recommended protocols involving:^12^

- Biofilm control, periodontal probing, and radiographic assessment before institution of orthodontics
- Ongoing plaque and inflammation control including six-monthly probing, and annual radiographic examinations during treatment; and
- Regular biofilm control and annual periodontal probing and radiographic examinations following the completion of active treatment.

Orthodontics is typically deferred until active inflammation has been resolved; moreover, adequate oral hygiene is a prerequisite for treatment. In the absence of significant periodontal inflammation or poor hygiene, treatment is generally compatible with periodontal health.^13^ Notwithstanding, clinically insignificant changes in periodontal parameters including increases in plaque and calculus debris, both quantitative and qualitative changes in oral microflora, and increased bleeding on probing (BOP) and PPD are typical. It is also noteworthy that characteristic differences in the microbiome exist in states of health and varying levels of disease.^14^

The standard approach to the management of orthodontic patients with periodontal inflammation has involved baseline stabilisation with ongoing maintenance before the institution of appliance therapy. Based on S3 guidelines on the management of Stage IV periodontitis,^5^ treatment should be deferred until the endpoints of periodontal therapy have been achieved with no sites with PPD in excess of 5 mm with BOP and with no sites in excess of 6 mm without BOP. Alternative approaches have been trialled including the use of orthodontics in parallel with periodontal intervention in order to improve access to periodontal defects for instrumentation.^15^ The latter approach was not proven to be beneficial with the standard of care supporting baseline periodontal stabilisation particularly in the resolution of moderate defects.^16^ A hiatus is therefore normally recommended in order to allow resolution of periodontal inflammation of three to six months following either non-surgical or surgical periodontal debridement. It is important to note, however, that the evidence base to support this recommendation is lacking.^2^

Regenerative surgical procedures are sometimes required at recalcitrant sites and due to anatomical complexity. Based on a recent clinical trial comparing orthodontic treatment commencing early (one month after surgery) or following a six-month interval, no short-term differences in periodontal parameters were noted. Furthermore, improved attachment gain was noted in the group having earlier orthodontics at 24-month follow-up suggesting that orthodontic treatment can commence as soon as one month following surgery.^16^^,^^17^

## Orthodontic planning in the periodontal patient

A history of severe loss of alveolar ridge height creates orthodontic challenges. This may have implications in relation to case selection, concerning treatment duration and regarding treatment objectives.^18^^,^^19^ Equally, thin periodontal phenotypes can be problematic to manage, although this will be discussed in an allied paper.^20^

### Case selection

**Fig. 2:**
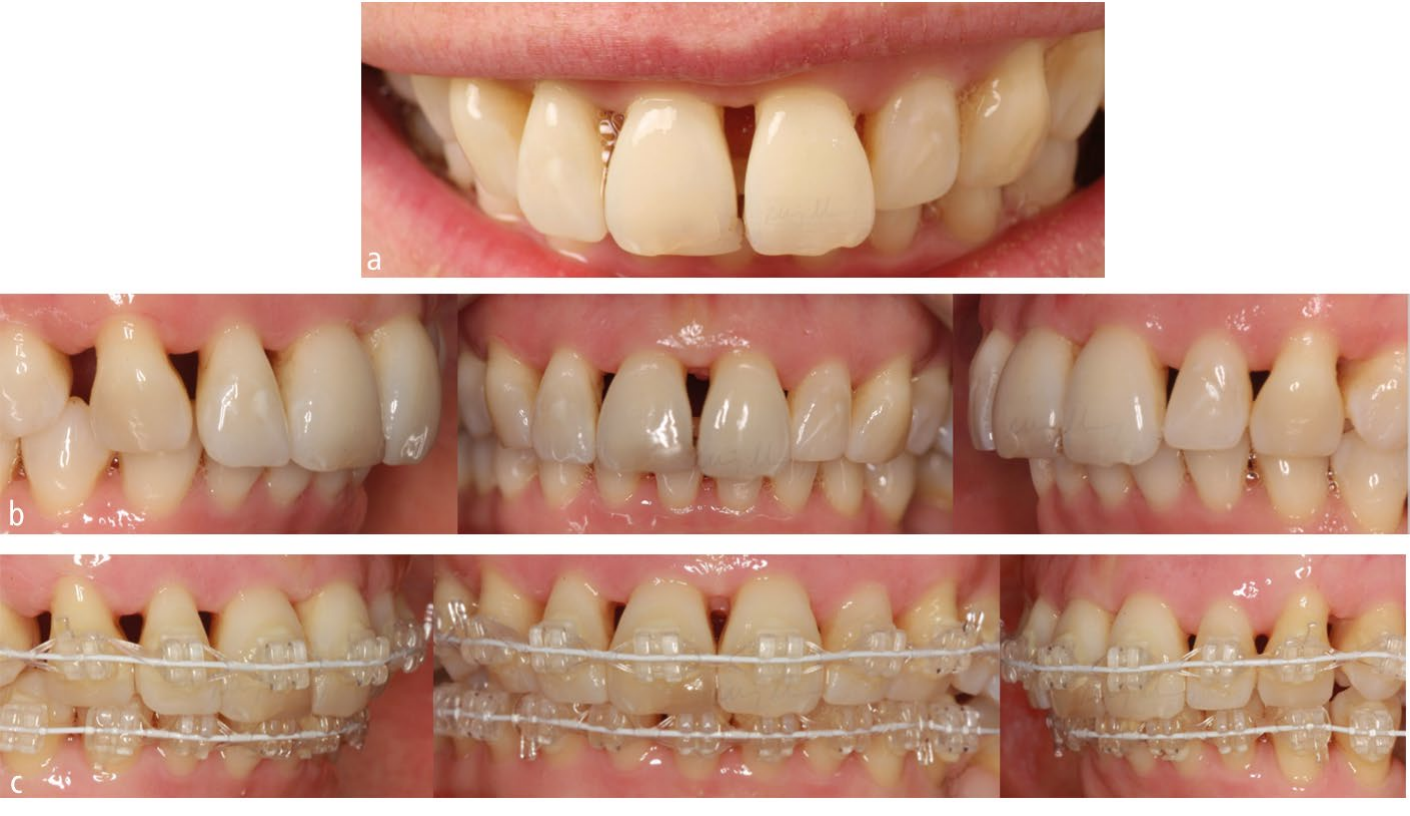
a, b) A 38-year-old woman had a history of previous orthodontics and tooth loss with spacing present in both arches c) Following stabilisation, fixed appliance-based treatment was undertaken. Inter-proximal reduction was planned in order to reduce the volume of open gingival embrasures; however, this was postponed until pre-existing space was consolidated

Case selection is important in orthodontics generally; however, the susceptibility of those with advanced periodontal breakdown to local and regional progression of the condition ensures that the risk to benefit ratio of intervention may be particularly unfavourable.^21^ As such, the treatment should only be considered in motivated patients capable of maintaining optimal levels of hygiene during active therapy and retention. Moreover, a targeted approach to treatment may be considered in order to assess the response to appliance therapy avoiding or in some cases postponing irreversible decisions including extractions or other forms of space creation (Fig. 2).

### Joint planning and ongoing management

Orthodontic treatment should only be initiated when periodontal care has been established and a favourable response to therapy observed. This includes biofilm control, baseline scoring and radiography with regular recall intervals^15^ varying from eight-weekly to six-monthly being defined based on the severity of the condition and risk profile. Moreover, periodontal input may be sensible in informing targeted orthodontic objectives in certain instances.

#### Tailored objectives

**Fig. 3:**
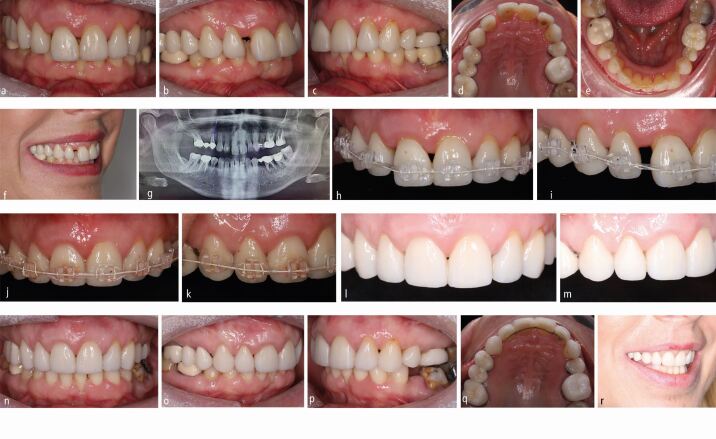
a, b, c, d, e, f, g) A 48-year-old woman presented with aesthetic concerns having had periodontal stabilisation. She had Grade IV periodontal disease with a heavily-restored dentition. h, i, j, k) A limited objective plan was devised involving the placement of an upper sectional fixed appliance, in isolation. Irregular spacing was closed early in treatment with contact point reshaping postponed. l, m, n, o, p, q, r) Overbite reduction was achieved by intrusion of the maxillary incisors promoting re-attachment

While comprehensive orthodontic treatment forms the mainstay of appliance therapy, a more nuanced approach may be more appropriate in those with Grade III or Grade IV involvement. As such, more severely affected or mobile teeth may be omitted from the appliances, as required. Equally, single-arch treatment may occasionally be considered (Fig. 3).

### Limit treatment duration

It is generally suggested that light forces should be used during orthodontics with the relationship between force levels and the rate of tooth movement being nonlinear.^22^ Notwithstanding, these forces are rarely measured and difficult to quantify.^23^ It is intuitive to expect that tooth movement is more efficient in those with more advanced periodontitis, although local anatomical issues may complicate this. It is important that anatomical complexities including atrophic ridges and bony deficiency are recognised and sustained, excessive forces are avoided.^24^

Shorter treatment duration has been linked with more favourable periodontal responses in these with advanced disease suggesting that prolonged therapy may risk inflammation due to plaque accumulation and changes in the microbiota.^25^ Moreover, it is conceivable that longer treatments might entail more significant root movements, which may predispose to local mobility transiently or indeed indefinitely, in turn risking further inflammation and attachment loss.

## Space creation and mechanics in the periodontal patient

### Phased intervention

While holistic correction of the malocclusion may well be planned, an iterative approach evaluating the overall hygiene and periodontal response is important in more severely compromised scenarios. Excessive mobility as well as deterioration in periodontal parameters including probing depth and bleeding on probing may warrant a more conservative orthodontic approach. Orthodontic objectives may require ongoing review with interventions phased, for example, by consolidating pre-existing space before creating additional space with extractions or inter-proximal reduction. This conservative approach may negate the possibility of inducing a deterioration in the periodontal condition during treatment.

### Space creation

The appropriate timing and titration of space creation is particularly important in patients with periodontitis. Excessive arch lengthening due to inadequate space creation carries associated risks in terms of instability, impaired function and disturbance of the roots from the alveolar housing. Equally, the generation of excessive space may dictate a prolonged space closure phase risking lengthening of treatment and application of excessive force for extended periods. Where extractions are planned, the possible aesthetic requirement for inter-proximal reduction should be factored. There is also a higher risk of reopening of extraction space in adult patients and those with compromised periodontal support.^26^

#### Inter-proximal reduction

**Fig. 4:**
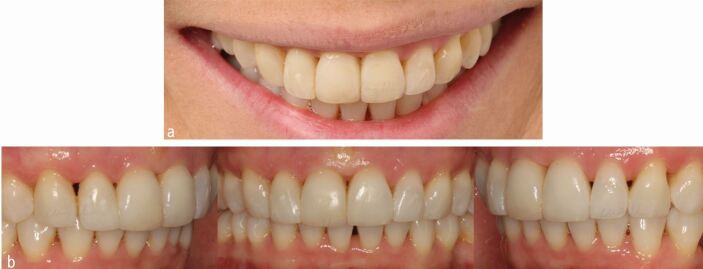
a, b) Adjunctive direct composite restorations were placed on the maxillary incisors to obscure open gingival embrasures following the completion of orthodontic treatment (earlier images in Figure 2). A fixed maxillary bonded retainer was placed directly following the restorative work

Inter-proximal reduction (IPR) may offer sufficient space in order to achieve orthodontic objectives. However, IPR may also be useful in limiting the volume of gingival embrasures in affected patients enhancing aesthetics, although adjunctive restorative procedures can be considered in order to address any residual issues (Fig. 4).

### Orthodontic appliances and mechanics

**Fig. 5:**
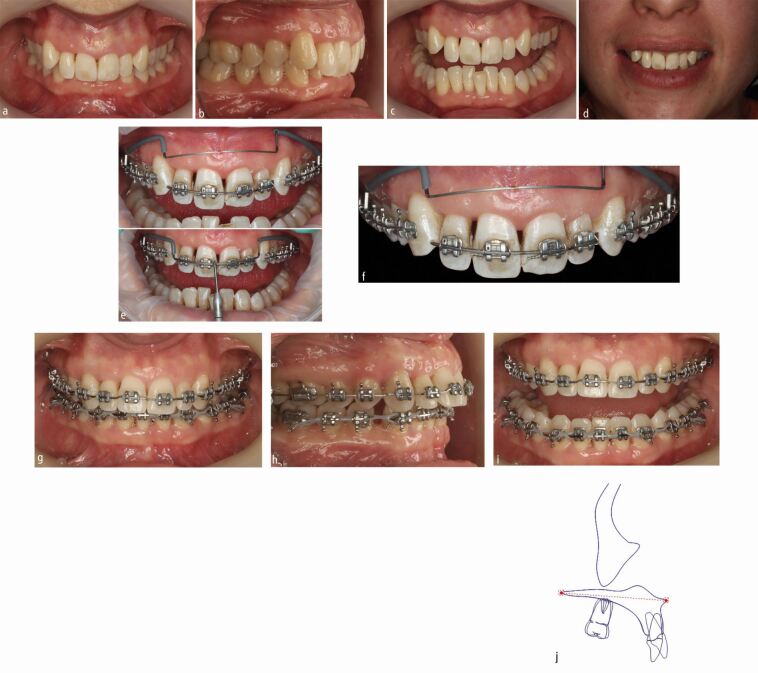
a, b, c, d) An adult woman with a Class II division 2 type malocclusion planned for mandibular advancement surgery with loss of a lower central incisor. e, f) Preferential intrusion of the maxillary incisors was undertaken with a 3-piece Rickett's utility arch permitting overbite reduction reducing excessive tooth maxillary incisal display while also permitting improvement in periodontal attachment. g, h, i) Light local interproximal reduction was undertaken concurrently to minimise open gingival embrasures. j) Regional maxillary superimposition highlights the extent of the maxillary incisal intrusion (2-3 mm) allied to maxillary incisal inclination change

A theoretical advantage of aligners may exist in terms of improved plaque and gingival indices.^27^ It is important to note, however, that both approaches are potentially compatible with periodontal health. Equally, a high prevalence of open gingival embrasures in the maxillary central incisor region (35% versus 18%) has been observed following treatment with aligners relative to fixed appliances.^28^ Moreover, it is imperative that both are used efficiently in order to achieve planned objectives. Local reattachment is thought to relate to intrusion of teeth within the periodontal apparatus with proclination more likely to induce open embrasure spaces. As such, tailored mechanics in order to limit unwanted extrusion or to facilitate true intrusion can be deployed (Fig. 5).

## Retention in the periodontal patient

Optimal retention is required in order to mitigate post-treatment changes due to orthodontic relapse and maturation. The approach to retention is often synonymous with that deployed in the general orthodontic cohort; however, a premium on maintenance and regular recall exists. A reduced periodontal support may predispose to rapid relapse; as such, the combined use of fixed and removable retention may offer a higher level of predictability.^29^ The impact of differential mobility, which may predispose to adhesive failure and fracture of fixed retainers should be considered both before and following appliance therapy.

## Conclusions

The benefit of orthodontic treatment in the overall management of advanced periodontitis is increasingly recognised. Conversely, it is important that periodontal inflammation is addressed before the commencement of active orthodontic treatment with appliance therapy risking qualitative and quantitative changes to the oral microbiota. Detailed interactions between the orthodontist, periodontist and general dentist are required to safeguard dental health and to produce optimal treatment outcomes involving a tailored approach to objective setting, treatment chronology, and mechanics. The latter should be flexible and subject to regular re-evaluation based on treatment response.
